# New Insights towards High-Temperature Ethanol-Sensing Mechanism of ZnO-Based Chemiresistors

**DOI:** 10.3390/s20195602

**Published:** 2020-09-30

**Authors:** Lesia Piliai, David Tomeček, Martin Hruška, Ivan Khalakhan, Jaroslava Nováková, Přemysl Fitl, Roman Yatskiv, Jan Grym, Mykhailo Vorokhta, Iva Matolínová, Martin Vrňata

**Affiliations:** 1 Department of Surface and Plasma Science, Faculty of Mathematics and Physics, Charles University, V Holešovičkách 2, 180 00 Prague 8, Czech Republic; lesiapiliai@gmail.com (L.P.); ivan.khalakhan@mff.cuni.cz (I.K.); jaroslava.novakova@mff.cuni.cz (J.N.); imatol@mbox.troja.mff.cuni.cz (I.M.); 2 Department of Physics and Measurements, University of Chemistry and Technology Prague, Technická 5, 166 28 Prague 6, Czech Republic; david.tomecek@centrum.cz (D.T.); martin1.hruska@vscht.cz (M.H.); premysl.fitl@vscht.cz (P.F.); 3 Institute of Photonics and Electronics, Czech Academy of Sciences, Chaberská 1014/57, 182 51 Prague 8, Czech Republic; yatskiv@ufe.cz (R.Y.); grym@ufe.cz (J.G.)

**Keywords:** near-ambient pressure XPS, ZnO nanorods, ethanol-sensing mechanism, acetaldehyde pathway, carbon contamination

## Abstract

In this work, we investigate ethanol (EtOH)-sensing mechanisms of a ZnO nanorod (NRs)-based chemiresistor using a near-ambient-pressure X-ray photoelectron spectroscopy (NAP-XPS). First, the ZnO NRs-based sensor was constructed, showing good performance on interaction with 100 ppm of EtOH in the ambient air at 327 °C. Then, the same ZnO NRs film was investigated by NAP-XPS in the presence of 1 mbar oxygen, simulating the ambient air atmosphere and O_2_/EtOH mixture at the same temperature. The partial pressure of EtOH was 0.1 mbar, which corresponded to the partial pressure of 100 ppm of analytes in the ambient air. To better understand the EtOH-sensing mechanism, the NAP-XPS spectra were also studied on exposure to O_2_/EtOH/H_2_O and O_2_/MeCHO (MeCHO = acetaldehyde) mixtures. Our results revealed that the reaction of EtOH with chemisorbed oxygen on the surface of ZnO NRs follows the acetaldehyde pathway. It was also demonstrated that, during the sensing process, the surface becomes contaminated by different products of MeCHO decomposition, which decreases dc-sensor performance. However, the ac performance does not seem to be affected by this phenomenon.

## 1. Introduction

ZnO is an intrinsic n-type semiconductor with a wide band gap (*E_g_* = 3.37 eV) and an exciton binding energy of 60 meV [1]. These properties, together with good chemical and thermal stability and low cost, make ZnO widely used in various fields and applications, including heterogeneous catalysis, photovoltaics and optoelectronics, and solar cells [2,3,4]. However, the most important application of ZnO is in chemiresistive gas sensors [5,6,7,8]. It was one of the first metal oxides reported as a promising material for the first gas sensor applications in the early 1960s [9]. Despite the fact that the ZnO-based gas sensors have been commercially used for the last three decades, the research on them is still far from being completed [10,11,12]. The research was particularly accelerated after the development of nanomaterials and nanotechnologies which demonstrated that the reduction in the grain size to nanometre dimensions significantly increases sensor sensitivity [13]. To date, the gas-sensing mechanism has not been fully explained. However, it is usually assumed that the change in sensor conductivity is connected with the chemisorption of atmospheric oxygen on the surface of the sensing layer [14]. Depending on the temperature, the oxygen extracts one or two free electrons from the surface, forming an electron depletion region there which reduces the total conductivity in the case of n-type semiconductors and increases it for p-type ones. Exposing the sensor to a reducing analyte (like ethanol) lowers the concentration of chemisorbed oxygen species (O_2_^−^, O^−^, and O^2^^−^) which return the electrons back to the surface and increase the conductivity [15]. In some cases, especially at a low temperature, the sensor conductivity may be related to the presence of molecular water or OH groups on the surface of the gas-sensitive material [16]. Sensor sensitivity is further related to many other parameters, including layer composition, morphology or the presence of dopants and catalysts. It has been demonstrated that nanostructuring can influence the thickness of the depleted region (Debye length), to be in tens of nanometers order for such metal oxides as SnO_2_ or ZnO [17].

A wide range of nanostructured ZnO films containing objects of different dimensions have been synthesized and tested as gas-sensing layers for gas sensors. Together with nanostructured ZnO films containing nanosized grains and nanocrystals, 1D and 2D nanostructured materials like nanorods (NRs), nanowires, nanosheets or nanobelts were reported to be effective for the detection of volatile organic compounds including ethanol (EtOH) and acetaldehyde (MeCHO). This broad topic is reviewed, e.g., in [18,19,20]. Partial studies dedicated to the detection of EtOH on ZnO NRs can be found in [21] or [22]. In the former case, the dc-response *S*_DC_ to 50 ppm of EtOH was 22 at 320 °C. In the latter case, the dc-responce *S*_DC_ to 1000 ppm of EtOH was 1.5 at 300 °C (for the definition of *S*_DC_, see Section 2.4). It is apparent that these results are significantly different. The detection of MeCHO exclusively on ZnO NRs is reported only in [23]. The authors achieved *S*_DC_ = 2.9 to 10 ppm of MeCHO at room temperature. Reports dedicated to the detection of EtOH or MeCHO on chemiresistors operating in ac-mode are rare. Certain conceptual approaches to the impedance measurement of sensors are discussed in [24]. In [25], the authors report the detection of ethanol vapours at a concentration of 0.7–5.0 ppm on ZnO nanorods at a working temperature of approximately 400 °C. The nanorods were grown via a hydrothermal route directly on a multielectrode chip. By employing impedance measurements in tandem with signal processing by linear discriminant analysis, it is possible to distinguish vapours of various alcohols even in the sub-ppm range.

As mentioned above, the sensing mechanism of EtOH by ZnO remains partially unclear. One problem is that there are no direct experimental results evidencing the oxygen chemisorption in situ on the ZnO (or other metal oxide) surface at the sensor working conditions [26]. Furthermore, the ethanol reaction pathway with the oxygen species on the ZnO at the usual sensor working temperature of 300–400 °C is frequently a matter of discussion. Based on the results from the reaction products’ observation (by gas chromatography and temperature-programmed desorption techniques), two competitive pathways were proposed. The first one assumes the reaction of EtOH with chemisorbed oxygen producing MeCHO and water [10,27]:

EtOH(ad) + O^−^ (ad) → CH_3_CHO + H_2_O + e^−^ (1)

EtOH(ad) + O^2−^ (ad) → CH_3_CHO + H_2_O + 2e^−^ (2)

The second pathway supposes C-O bond scission leading to EtOH dehydration and ethylene formation, which then interacts with the oxygen ions, generating water and carbon dioxide [28,29]:

EtOH(ad) → C_2_H_4_(ad) + H_2_O (3)

C_2_H_4_(ad) + 3O_2_^2−^(ad) → 2CO_2_ + 2H_2_O + 6e^−^ (4)

However, neither of these mechanisms account for the possible change in the sensing layer stoichiometry during interaction with EtOH, or its deactivation by different strongly bonded carbonaceous species (coke, acetates) that may be formed as a result of the ethanol decomposition. Therefore, to resolve the mechanism, the utilization of an in situ/operando technique that provides information about and the sensing layer stoichiometry and various intermediate species on the surface under operational conditions is essential.

Near-ambient-pressure X-ray photoelectron Spectroscopy (NAP-XPS) is a technique that allows chemical investigation of solid surfaces in the presence of gases and vapours and provides important information on the reaction intermediate species appearing on the surface during different chemical reactions [30,31,32]. We recently utilized NAP-XPS for in situ investigation of the gas-sensing mechanism of nanostructured SnO_2_- and CuO_x_-based gas sensors [33,34]. In this work, we applied NAP-XPS for the investigation of the EtOH- and MeCHO-sensing mechanisms of a ZnO NRs-based chemiresistor. First, sensing performance towards 100 ppm of EtOH and MeCHO in the ambient air at 327 °C was evaluated using a gas sensor with the ZnO NRs-sensing layer. Then, a ZnO NR film on a silica substrate (fabricated in the same way) was investigated by NAP-XPS in various gas/vapour mixtures, simulating its normal operating conditions. The partial pressure of the analytes was 0.1 mbar, as it corresponds to the concentration of 100 ppm under ambient pressure. To improve the understanding of the mechanism, the influence of water vapour was also investigated.

## 2. Materials and Methods

### 2.1. Synthesis of ZnO NRs and Fabrication of Sensors

**Figure 1:**
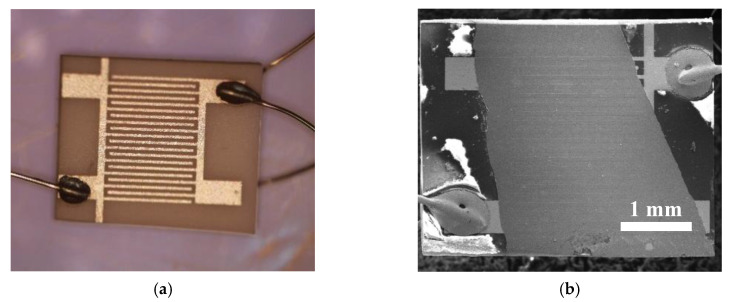
Optical microscope image of the alumina-sensing platform with Pt electrodes (**a**) and SEM image of the ZnO NRs prepared on it (**b**).

The ZnO NRs were grown on seeding layers by chemical bath deposition (CBD) from equimolar aqueous solutions consisting of 50 mM zinc nitrate hexahydrate and hexamethylentetramine at 95 °C for 2 h [35]. The NRs were prepared on a silicon substrate for the NAP-XPS measurements, while for the investigation of the sensing properties, alumina substrates with Pt electrodes were used (Figure 1). The seed layer was deposited by pulsed laser deposition. Deposition was carried out by a laser *Quantel Brilliant* (wavelenght 266 nm, pulse duration 4 ns, repetition rate 10 Hz). The energy of laser pulses was 45 mJ. The target was made from ZnO with 5N purity (pressed and then sintered in a furnace). The system was evacuated to high vacuum conditions and the deposition was carried out in 20 Pa of oxygen at room temperature. The target substrate distance was 5 cm. The total number of laser pulses was 300. The AFM image of the seed layer on the silicon substrate is presented in Figure S1 of the Supporting Information.

### 2.2. Morphological and Structural Characterizations

The morphology and structure of the ZnO NRs were examined by scanning electron microscopy (SEM) using a *Tescan MIRA 3* microscope operating at 30 keV electron beam energy and by high-resolution transmission microscopy (HRTEM) using a 200 kV *JEOL 2100FEG UHR* microscope with a Scherzer resolution of 0.19 nm. The TEM images were recorded by a charge-coupled device camera and analyses of the results were performed using the *Digital Micro-graph* software.

### 2.3. In Situ NAP-XPS Characterizations

The NAP-XPS measurements were performed using a lab-based NAP-XPS system (*SPECS Surface Nano Analysis, GmbH Germany*) equipped with the monochromatized Al Kα X-ray source, hemispherical electron energy analyser and specially designed NAP cell. A detailed description of this system can be found in [33]. Investigations into the sensor interaction with a low concentration of ethanol were performed as follows: first, the XPS spectra were acquired at 327 °C in 1 mbar of oxygen, mimicking the ambient atmosphere without a reducing agent; then, the same sets of spectra were measured in the O_2_/EtOH mixture, prepared by increasing the total gas pressure in the NAP cell to 1.1 mbar by adding ethanol vapour. The O_2_/EtOH/H_2_O mixture was created by adding 0.1 mbar of water vapour to the EtOH/O_2_ mixture. Finally, the same set of spectra was measured in the presence of the O_2_/MeCHO atmosphere prepared inside the NAP cell in a similar way (by the increase in the total gas pressure to 1.1 mbar and by the addition of 0.1 mbar of acetaldehyde vapour). During all NAP-XPS measurements, the Zn 2p, Zn LMM, O 1s and C 1s spectra were recorded at a constant pass energy of 20 eV and a photoelectron emission angle of 0°, with respect to the sample normal. The spectral components in the core-level spectra were fitted with a Voigt profile after subtracting the Shirley background using the *KolXPD*-fitting software.

### 2.4. Instrumentation and Evaluation of Sensor Measurement in Dc- and Ac- Mode

**Figure 2:**
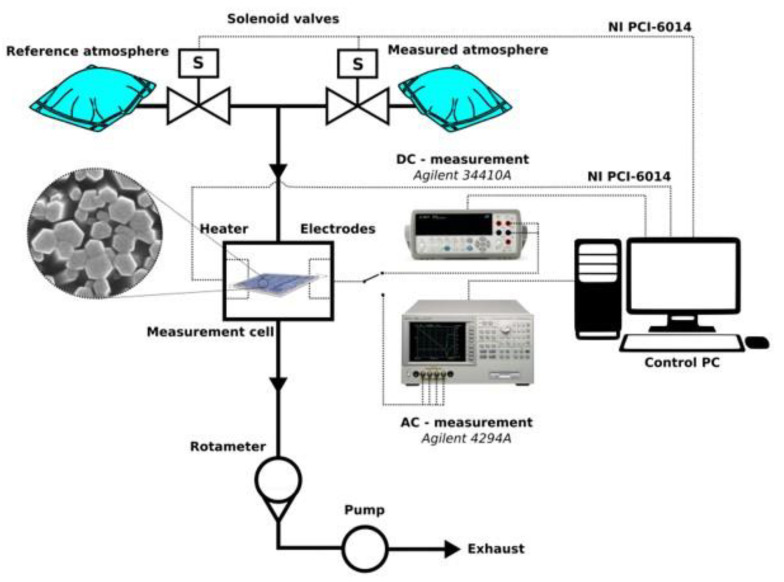
Block diagram of the station for measuring the chemiresistor response.

DC measurements were conducted with an *Agilent 34410A* in a sensor evaluation system described in [36]. The AC ones (impedance measurements) were performed with an *Agilent 4294A* impedance analyzer in a system depicted in Figure S2 of SI. The impedance measurements were carried out in two-wire connection in the frequency range of 40 Hz–100 MHz (at 800 frequency points in logarithmic scale). The amplitude of a testing signal was set to 10 mV with no bias voltage. Both DC and AC measurements were done for four distinct analyte mixtures (EtOH; MeCHO; EtOH + H_2_O; MeCHO + H_2_O) with the concentration of 100 ppm for each component (the rest of the mixture was synthetic air at 1 bar pressure). The gas flow was set to 25 mL/min and sensor temperature to 327 °C. The block diagram of the station for measuring the chemiresistor response in the DC and AC modes is presented in Figure 2.

First, the sensors were stabilized at their operating temperature for 2 h. Then, they were alternately exposed to analyte and reference mixtures at 1 bar pressure. The reference mixture was pure synthetic air without any analyte. The exposure to analyte mixtures lasted 10 min. The exposure to the reference mixture took 30–60 min depending on the speed of sensor recovery.

Sensor responses were evaluated using the following quantities: *S*_DC_, *S*_AC40Hz_ and *S*_PA-MAX_. *S*_DC_ was defined as a ratio of corresponding pre-exposure and post-exposure resistances (*S*_DC_ = *R*_air_/*R*_analyte_). The impedance data were processed as follows: (i) Nyquist plots (i.e., imaginary part of complex impedance Im (*Z*) vs. real part complex impedance Re (*Z*) with the frequency of measuring signal as a parameter) were constructed. (ii) The phase angles *Θ* of complex impedance of the sensor in “pure” synthetic air (*Θ*_air_ = arccos [Re(*Z*_air_)/|*Z*_air_|]) and complex impedance of the same sensor in analyte (*Θ*_analyte_ = arccos [Re(*Z*_analyte_)/|*Z*_analyte_|]) were calculated. The symbol |*Z*|denotes impedance modulus, which means |*Z*|= [Re^2^(*Z*) + Im^2^(*Z*)]^1/2^. (iii) Phase-angle sensitivity *S*_PA_ was defined as a difference in the phase angles in synthetic air and in analyte, respectively, i.e., *S*_PA_ = *Θ*_air_ − *Θ*_analyte_. It is clear that *Z*, *Θ* and *S*_PA_ are frequency-dependent quantities. (iv) The parameter *S*_PA-MAX_ is the maximum value of *S*_PA_ achieved for a sensor. (v) The parameter *S*_AC40Hz_ is a ratio of the real parts of the corresponding impedances in air and in analyte at 40 Hz, i.e., *S*_AC40Hz_ = Re (*Z*_air_)/Re (*Z*_analyte_). Further information concerning AC measurements is available in [37,38].

## 3. Results and Discussion

### 3.1. Structural and Morphological Analysis

**Figure 3:**
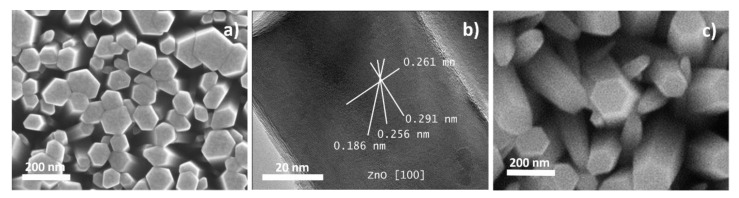
SEM (**a**) and TEM (**b**) images of the ZnO NRs grown on the silicon substrate and (**c**) SEM image of the ZnO NRs prepared on the sensing platform.

Figure 3a presents the SEM micrograph of the ZnO NRs grown on the silicon substrate for the NAP-XPS measurements. The image shows a compact array of vertically aligned, single-crystalline ZnO NRs with a diameter varying within the 50−150 nm range. The TEM image of the top part of a single ZnO NR is presented in Figure 3b. It confirms the crystallinity of ZnO NRs with a hexagonal wurtzite structure. The NRs prepared on the alumina sensor substrates for investigation of the EtOH-sensing properties (depicted in Figure 3c) had the same size, but more random orientation due to the greater substrate roughness.

### 3.2. NAP-XPS Analysis

**Figure 4:**
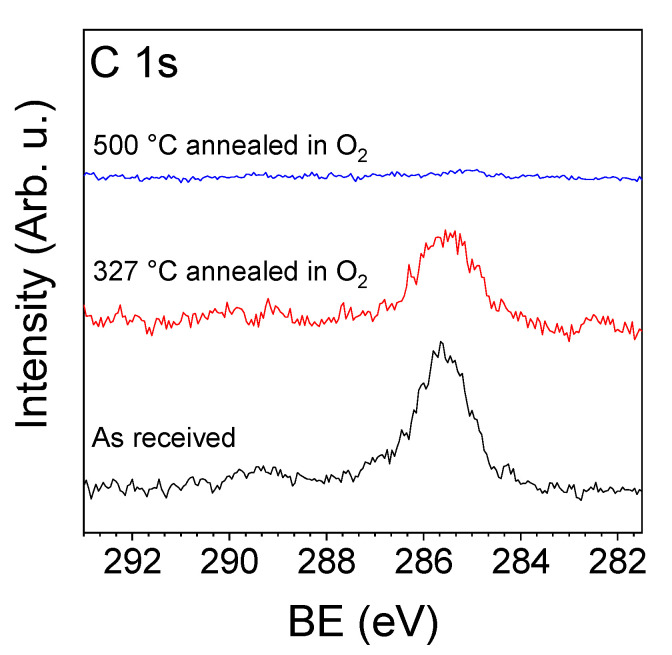
NAP-XPS spectra of the C 1s core-level acquired from the as-received ZnO NRs, after annealing in 5 mbar O_2_ at 327 °C and after annealing in 5 mbar O_2_ at 500 °C.

The XPS measurements of the as-prepared ZnO NRs done in the presence of 1 mbar of oxygen at room temperature showed a relatively high amount of carbon contamination on the surface (Figure 4). After heating the sample to a sensor working temperature of 327 °C in 5 mbar of oxygen, the amount of surface carbon decreased by only about 30%. Thus, prior to the measurements, a special sample cleaning protocol was applied, which included the sample annealing at 500 °C for about 60 min in 5 mbar of O_2_ with a further decrease in the oxygen pressure and sample temperature to 1 mbar and 327 °C, respectively. After this, the carbon contamination decreased to about 5% of the initial amount and the sensor was considered to be cleaned. However, this indicates that the carbon contamination must also be present on the surface of a real working sensor and should be taken into account when considering the sensing mechanism and sensor performance. The cleaned sensor was then exposed to the O_2_/EtOH, O_2_/EtOH/H_2_O and O_2_/MeCHO gas atmospheres at 327 °C in order to understand the processes taking place on the surface of the ZnO NRs-based sensor while sensing EtOH or MeCHO molecules. Before each exposure, the sample was also cleaned, but it was enough to anneal it at only 327 °C in the presence of 5 mbar O_2_ to remove all carbon.

**Figure 5:**
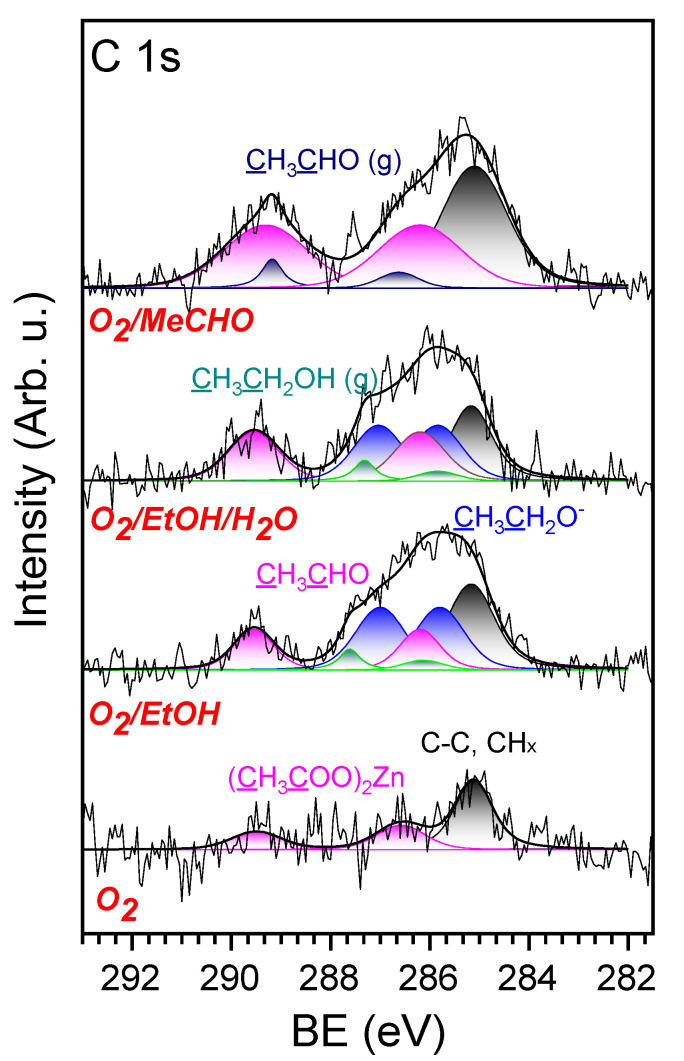
NAP-XPS spectra of the C 1s core-level acquired on the ZnO NRs-based sensor in the presence of O_2_, O_2_/EtOH, O_2_/EtOH/H_2_O and O_2_/MeCHO at 327 °C.

The C 1s spectra measured in the presence of O_2_, O_2_/EtOH, O_2_/EtOH/H_2_O and O_2_/MeCHO are presented in Figure 5. The C 1s spectrum acquired from the cleaned ZnO NRs in the presence of oxygen (the bottom spectrum) showed some small amount of carbon contamination, with the main peak at about 285.2 eV (black peak). This peak can be assigned to amorphous carbon and different CH_x_ species adsorbed on the surface of ZnO [39]. The two peaks at 286.5 and 289.4 eV (pink color peaks) most probably belong to the methyl and carboxylate groups of surface acetates [40], which appeared on the surface during NRs synthesis. The peak at 289.4 eV could also originate from carbonates that may be formed after high-temperature annealing of the contaminated ZnO surface in oxygen. Sample exposure to the O_2_/EtOH mixture led to significant changes in the C 1s spectrum (second spectrum from the bottom in Figure 5). The peaks assigned to the surface acetates doubled, and position of the methyl peak shifted to a lower binding energy (BE) by 0.3 eV. The peak assigned to amorphous carbon and CH_x_ groups at 285.2 eV also increased. In addition, two equal peaks at about 285.6 and 287 eV (blue peaks), assigned to the methyl and alxoxy groups of ethoxy (the intermediate product of ethanol dehydrogenation), respectively, and two small peaks at about 287.6 and 286.1 eV of gas phase EtOH (green peaks) appeared in the spectrum. The intensity and shape of the EtOH gas peaks, as well as the distance between them, were determined on the basis of a reference measurement of an O_2_/EtOH mixture with no sample inside the NAP cell (see Figure S3 of SI). It is worth noting that the increase in the peaks’ intensity at 289.4 and 286.5 eV, together with the shift of the last by 0.3 eV to lower BE (from 286.5 eV to 286.2 eV), is most probably related to the presence of MeCHO on the surface. Indeed, MeCHO contains a formyl (aldehyde) group with a double C = O bond, giving rise to an XPS peak at about 289–290 eV and a methyl group producing an XPS peak at a slightly lower BE of 285–286 eV compared to the surface acetates [41]. Adding water to the O_2_/EtOH mixture (second spectrum from the top in Figure 5), resulted in a slight increase in the MeCHO signals and a decrease in the ethoxy and amorphous carbon peaks. The C 1s spectrum measured in the presence of the O_2_/MeCHO mixture (the top spectrum in Figure 5) consisted of the already-observed peaks of adsorbed MeCHO and amorphous carbon, and two new peaks at about 289 and 286.4 eV (dark blue), which are the formyl and methyl features of gas-phase MeCHO. The intensity and shape of these gas-phase peaks, as well as the distance between them, were found from the reference NAP-XPS measurement of O_2_/MeCHO gas mixture (Figure S3 of SI).

**Figure 6:**
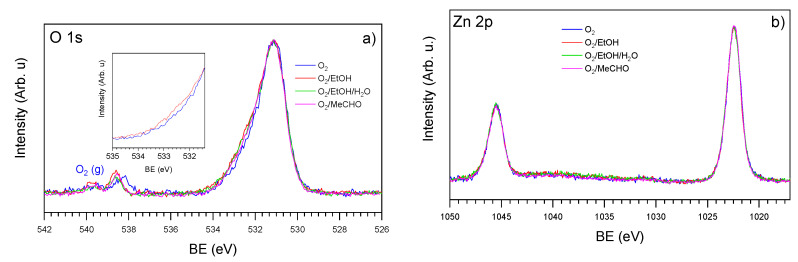
NAP-XPS spectra of O 1s (**a**) and Zn 2p (**b**) acquired from the ZnO NRs-based sensor in the presence of O_2_, O_2_/EtOH, O_2_/EtOH/H_2_O and O_2_/MeCHO at 327 °C.

The corresponding O 1s, Zn 2p and Zn LMM spectra acquired in the presence of O_2_, O_2_/EtOH, O_2_/EtOH/H_2_O and O_2_/MeCHO are presented in Figure 6 and Figure S4 of SI. The O 1s spectra showed slight changes depending on the exposing atmosphere. This is mainly because of the strong oxygen signal originating from bulk ZnO, with the main state at about 531 eV. This peak is so intense that the low oxygen signal originating from the adsorbate is almost undetectable by XPS. The inset in Figure 6a, demonstrating the difference between O 1s spectra taken in O_2_ and O_2_/EtOH atmospheres, shows an increase in oxygen signal in the 531.5–533 eV region. In this region, the oxygen signals from different hydrocarbons containing oxygen, H_2_O and OH usually appear [42]. Similar signal increases can be observed in the case of O_2_/EtOH/H_2_O and O_2_/MeCHO atmospheres. However, these contributions are so small that it is impossible to make any quantitative analysis or to fit them. Compared to the oxygen spectra, the corresponding Zn 2p spectra contained even smaller changes (Figure 6b). In all cases, there was a doublet, with the position of the main peak at about 1022 eV corresponding to the Zn^2+^ ions of bulk ZnO [43]. Comparison of the spectra after their normalization to the same height did not show any difference in their shape, except for a shift of about 0.15 eV to higher binding energy for the spectrum acquired in the pure oxygen atmosphere. As this shift was noticed for the whole set of spectra (Zn 2p, Zn LMM, O 1s and C 1s spectra) collected in oxygen, we attributed it to the band-bending effect raised by oxygen chemisorption that occurs at 327 °C [44]. For easier comparison, the effect of band bending was eliminated by shifting the whole set of spectra measured in oxygen by 0.15 eV to a higher BE prior to the spectra processing. The Zn LMM Auger spectra were acquired together with the Zn 2p spectra and presented in Figure S4 of SI. These spectra resembled typical Auger spectra of ZnO with the main peak at about 988 eV [43], regardless of the exposing atmosphere.

The NAP-XPS results presented above supported the theory of an MeCHO reaction pathway for the EtOH reaction with the chemisorbed oxygen species described in the introduction. We could not detect any signal related to ethylene with a double C = C bond usually appearing at 284–284.2 eV [45], which rules out the ethylene pathway at this temperature. It is apparent that the EtOH gas-sensing mechanism includes the formation of the ethoxy group that is an intermediate product of MeCHO formation via two-step EtOH dehydrogenation. (The process includes an O-H bond scission forming the ethoxy group in the first step. The second hydrogen detaches in the second step, and the carbon skeleton forms MeCHO). The detached hydrogens interact with the chemisorbed oxygen species, forming H_2_O. However, the more intense peak in amorphous carbon at 285.2 eV in case of the O_2_/MeCHO measurements points to further decomposition of the MeCHO molecules that, in turn, could decrease sensor performance. This may explain the lower response of the ZnO sensor to MeCHO, as discussed in the next section. Moreover, it looks as if water on the surface of ZnO NRs partially blocks the MeCHO decomposition, leading to the increase in its concentration on the surface and a slight decrease in amorphous carbon formed as a result of MeCHO decomposition. However, as shown below, this has a very low influence on sensor performance.

### 3.3. Response of Sensor in Dc- and Ac-Modes

**Figure 7:**
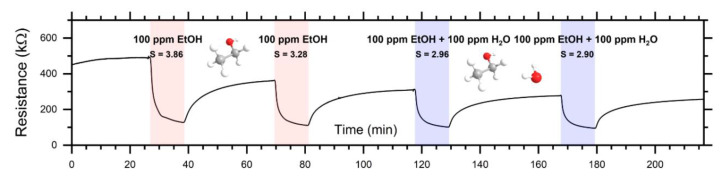
Responses of the ZnO-nanorod-based chemiresistor to EtOH and EtOH/H_2_O vapours. The sequence of atmospheres was as follows: reference air → 100 ppm EtOH → reference air → 100 ppm EtOH → reference air → 100 ppm EtOH + 100 ppm H_2_O → reference air → 100 ppm EtOH + 100 ppm H_2_O → reference air.

**Figure 8:**
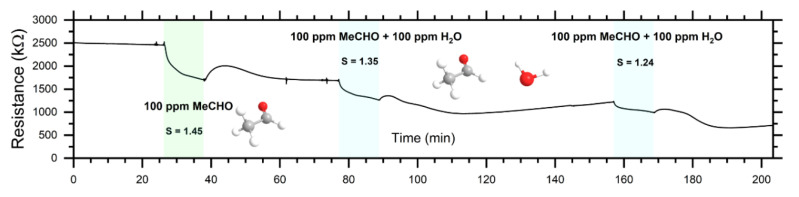
Responses of the ZnO-nanorods based chemiresistor to MeCHO and MeCHO/H_2_O vapours. The sequence of atmospheres was: reference air → 100 ppm MeCHO → reference air → 100 ppm MeCHO + 100 ppm H_2_O → reference air → 100 ppm MeCHO + 100 ppm H_2_O → reference air.

**Figure 9:**
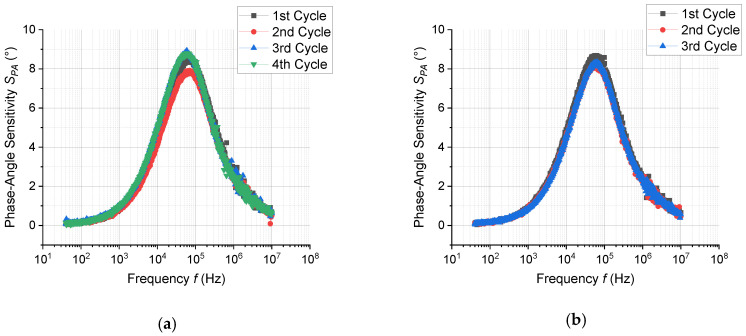
The dependence of phase-angle response vs. frequency for 100 ppm EtOH (**a**); 100 ppm EtOH + 100 ppm H_2_O (**b**). The reference atmosphere was synthetic air.

**Figure 10:**
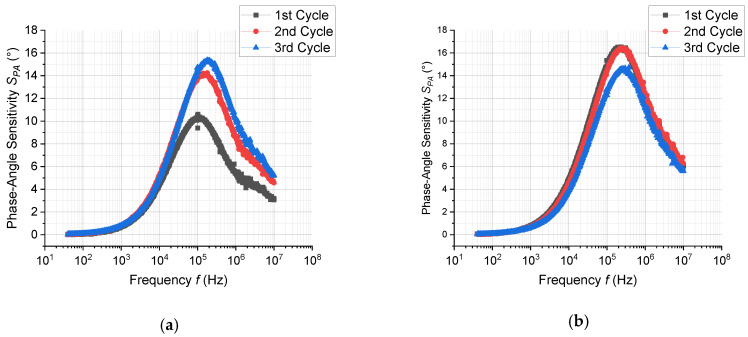
The dependence of phase-angle response vs. frequency for 100 ppm MeCHO (**a**); 100 ppm MeCHO + 100 ppm H_2_O (**b**). The reference atmosphere was synthetic air.

To investigate the sensing properties of ZnO-nanorods in macroscale, the prepared sensor devices (chemiresistors) were measured in both dc- and in ac-mode. Figure 7 depicts a dc-response to EtOH and EtOH/H_2_O, while Figure 8 depicts the dc-response to MeCHO and MeCHO/H_2_O. Figure 9 and Figure 10 present certain results extracted from ac-responses, i.e., the dependence of phase-angle responses on the frequency of a measuring signal for EtOH and EtOH/H_2_O (Figure 9) or MeCHO and MeCHO/H_2_O (Figure 10). Table 1 summarizes the important sensor parameters evaluated from Figure 6, Figure 7, Figure 8, Figure 9 and Figure 10, namely, dc-responses, ac-responses at 40 Hz and maximum phase-angle responses *S*_PA-MAX_. Both instrumentation of the measurement and sensor parameters evaluation were carried out according to the procedures described in the experimental part.

In the following text, sensor responses are analyzed first, and, secondly, they are correlated with the phenomena revealed by the NAP-XPS spectroscopy of corresponding systems. Analysis of the sensor responses resulted in the following observations:

(i) ZnO behaves like an n-type semiconductor—both reducing analytes (EtOH and MeCHO) decrease either its resistance or its impedance modulus (see Figure 7 and Figure 8);

(ii) The responses to EtOH and EtOH/H_2_O are more stable than the responses to MeCHO and MeCHO/H_2_O. This is especially apparent when comparing the stability of the sensor baseline during the dc-measurement (cf. Figure 7 and Figure 8). Within the ac-measurements, this fact is manifested by a significant drift in *S*_PA_ in time for MeCHO (cf. Figure 9 and Figure 10);

(iii) While the *S*_DC_ values are higher for EtOH and EtOH/H_2_O, *S_AC_*_40Hz_ and *S*_PA-MAX_ are greater for MeCHO and MeCHO/H_2_O, i.e., EtOH is easily detectable by a dc-measurement and MeCHO by an ac-measurement;

(iv) The effect of water on chemiresistor responses is relatively weak at 327 °C;

(v) The magnitude of the dc-response is somewhat decreased in each subsequent step (see Figure 7 and Figure 8).

The observation (i) is “trivial”, as ZnO has been many times reported to exhibit n-type conductivity. The observations (ii), (iii) and (v) are apparently connected with the amount of MeCHO and amorphous carbon on the surface of ZnO NRs. As shown from the NAP-XPS measurements, the MeCHO molecules are formed as a result of the EtOH dehydrogenation and obviously undergo further decomposition. The NAP-XPS spectra confirm that the amount of amorphous carbon and CH_x_ species on the ZnO surface gradually increases during the measurement. It also shows that this increase is significantly higher in the case of MeCHO. As a result, we can formulate the following hypothesis: When detecting EtOH or EtOH/H_2_O, the surface of ZnO is only slightly decorated with amorphous carbon, oxygen species chemisorbed on the ZnO surface react with hydrogen atoms from EtOH, ethoxy and MeCHO dehydrogenations and the sensor response is primarily determined by the exchange of electrons between chemisorbed oxygen and zinc oxide nanorod. Thus, the drift component of the current is affected, which results in a higher response when measuring in dc-mode. In this case, the concentration of produced MeCHO molecules probably remains below some critical amount, as it has enough time to desorb from the surface and only a very slow accumulation of the surface carbon contamination takes place. On the other hand, when detecting MeCHO, the amount of the carbon species on ZnO surface is higher and the concentration of the active sites for oxygen species chemisorption is smaller. Instead, organic molecules containing dipoles are present: acetaldehyde and intermediates of the acetaldehyde oxidation. Consequently, the displacement component of the current is affected, which implies a higher response of the sensor in ac-mode. Finally, a certain decrease in the dc-response magnitude stems from the gradually increasing surface contamination.

Our results concerning the detection of EtOH and MeCHO can be compared to observations published in [46]. In that study, the nanoparticles of ZnO were synthesized by a hydrothermal process from zinc hydroxide and trisodium citrate, i.e., the preparation method was similar. The dc-sensitivity *S*_DC_ achieved for 100 ppm of ethanol at 300 °C was 7.5 and 5.0 to 100 ppm of MeCHO. The magnitude of dc-responses was higher than that of our sensors (*S*_DC_ = 3.6 to 100 ppm of EtOH at 327 °C and *S*_DC_ = 1.5 to 100 ppm of MeCHO at 327 °C—see Table 1), but the trend was the same. In both cases, the dc-responses to EtOH were greater than the responses to MeCHO. It is notable that the authors explain the response of ZnO to EtOH and MeCHO only by their interaction with chemisorbed oxygen species, resulting in the total oxidation of these analytes to CO_2_ and H_2_O. However, they neither carriedac-measurement nor analyzed surface deposits on the ZnO nanoparticles. Contrary to their conclusion, we proved that surface species play an important role in influencing the detection mechanism.

The influence of water on the sensitivity of ZnO nanoparticles to ethanol at a high temperature was published in [47]. It was shown that the response to 300 ppm ethanol (measured at 400 °C) was slightly decreased (by 3−14%) in the atmosphere containing 56% relative humidity, which is consistent with our observation (iv). The low influence of water on the sensitivity of ZnO-based chemiresistors at high temperatures is most probably related to the rapid desorption of water molecules from ZnO surface. It was shown that the adsorption probability of 0.1 ML of water on ZnO surface at 330 °C approaches zero [48], which means a weak blocking of adsorption sites for oxygen and ethanol molecules.

## 4. Conclusions

This work presents the results of the in situ NAP-XPS investigation of the EtOH- and MeCHO-sensing properties of ZnO nanorods at high temperature. We demonstrated that the EtOH-sensing mechanism at high temperature (327 °C) is based on the dehydrogenation of EtOH molecules, leading to ethoxy and MeCHO, where the detached hydrogens interact with the chemisorbed oxygen, causing a sensor response. It was also shown that MeCHO further decomposes on the surface producing amorphous carbon and various CH_x_ species, which seemed to be responsible for the decay in dc-sensor detection performance. When detecting EtOH, the concentration of generated MeCHO molecules remained relatively low, so that it had enough time do desorb or to completely oxidize causing a relatively slow accumulation of the surface carbon contamination on the surface of ZnO, so the chemisorbed oxygen species could react with the detached hydrogen atoms, preserving the sensitivity. On the other hand, in the case of MeCHO-sensing, the carbon contamination on the surface is substantially higher and partially blocks the oxygen chemisorption on it. This phenomenon leads to the decrease in the dc-response magnitude and affection of the displacement component of the current, causing higher sensor responses in ac-mode.

## Figures and Tables

**Table 1:** Detection parameters of ZnO-nanorod based chemiresistors: dc-response (*S*_DC_); ac-response at 40 Hz (*S_AC_*_40Hz_); maximum phase-angle response *S*_PA-MAX_ and the corresponding frequency of the measuring signal. The reference atmosphere was synthetic air in all cases.

	Dc-Parameter	Ac-Parameter	
**Analyte**	*S*_DC_	*S_AC_*_40Hz_	*S*_PA-MAX_ [deg] at frequency [Hz]
EtOH (100 ppm)	3.6	1.5	8.3 at 6 × 10^4^ Hz
EtOH (100 ppm) + H_2_O (100 ppm)	2.9	1.5	8.5 at 6 × 10^4^ Hz
MeCHO (100 ppm)	1.5	2.2	12.5 at 2 × 10^5^ Hz
MeCHO (100 ppm) + H_2_O (100 ppm)	1.3	2.6	15.5 at 2 × 10^5^ Hz

## Supplementary Materials

The following are available online at https://www.mdpi.com/1424-8220/20/19/5602/s1, Figure S1: AFM image of the ZnO seed layer on silicon wafer by PLD, Figure S2: Photo of measurement apparatus used for the sensor response measurements in AC mode, Figures S3 and S4: Supplementary NAP-XPS spectra.
