# Supplementary material for: New Insights towards High-Temperature Ethanol-Sensing Mechanism of ZnO-Based Chemiresistors
Source: Sensors (Basel). 2020 Sep 30;20(19):5602. doi: 10.3390/s20195602 (PMC7582869; doi:10.3390/s20195602)
Supplement: Supplementary file 1 [file sensors-20-05602-s001.pdf]

Supporting Information

New Insights Towards High-Temperature Ethanol-Sensing Mechanism of ZnO-Based Chemiresistors

Lesia Piliai [1], David Tomeček [2], Martin Hruška [2], Ivan Khalakhan [1], Jaroslava Nováková [1], Přemysl Fitl [2], Roman Yatskiv [3], Jan Grym [3], Mykhailo Vorokhta [1,*], Iva Matolínová [1] and Martin Vrňata [2,*]

1 Department of Surface and Plasma Science, Faculty of Mathematics and Physics, Charles University, V Holešovičkách 2, 180 00 Prague 8, Czech Republic; lesiapiliai@gmail.com (L.P.); ivan.khalakhan@mff.cuni.cz (I.K.); jaroslava.novakova@mff.cuni.cz (J.N.); imatol@mbox.troja.mff.cuni.cz (I.M.)

2 Department of Physics and Measurements, University of Chemistry and Technology Prague, Technická 5, 166 28 Prague 6, Czech Republic; david.tomecek@centrum.cz (D.T.); martin1.hruska@vscht.cz (M.H.); premysl.fitl@vscht.cz (P.F.)

3 Institute of Photonics and Electronics, Czech Academy of Sciences, Chaberská 1014/57, 182 51 Prague 8, Czech Republic; yatskiv@ufe.cz (R.Y.); grym@ufe.cz (J.G.)

* Correspondence: vorokhtm@mbox.troja.mff.cuni.cz (Mykhailo Vorokhta); martin.vrnata@vscht.cz (Martin Vrňata)

Received: 31 August 2020; Accepted: 23 September 2020; Published: 30 September 2020

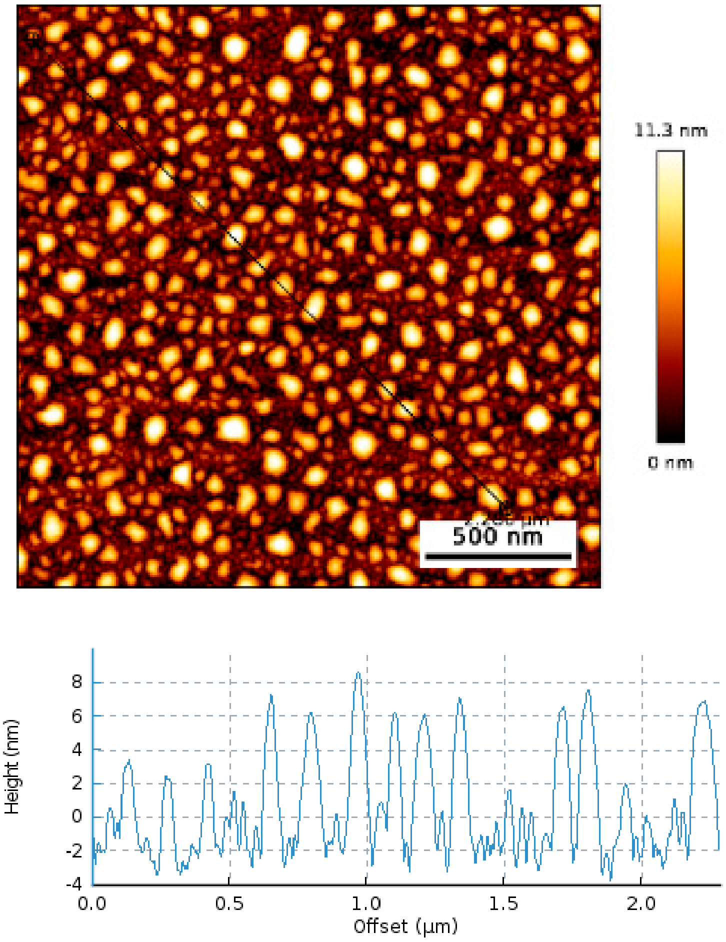

Figure S1. AFM image of the ZnO seed layer deposited on silicon wafer by PLD

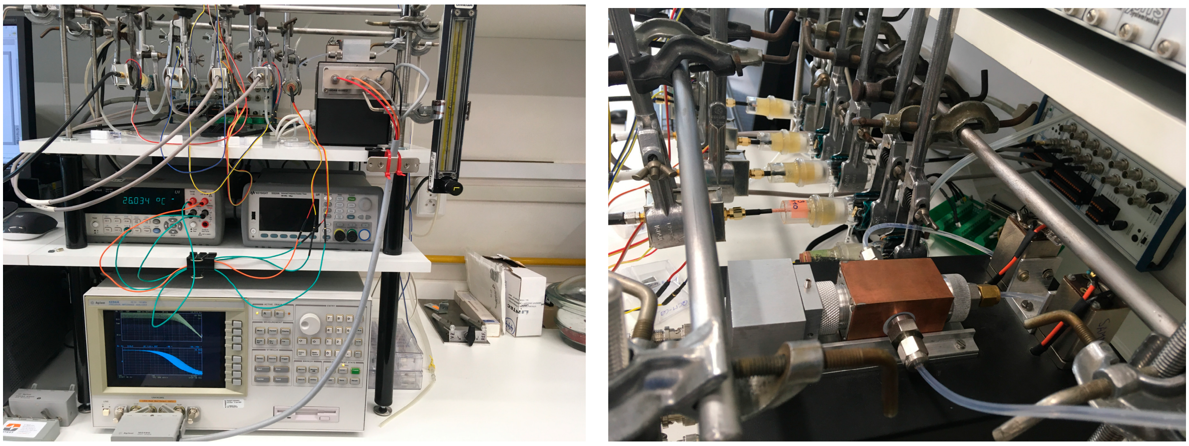

Figure S2. Photo of measurement apparatus used for the sensor response measurements in AC mode

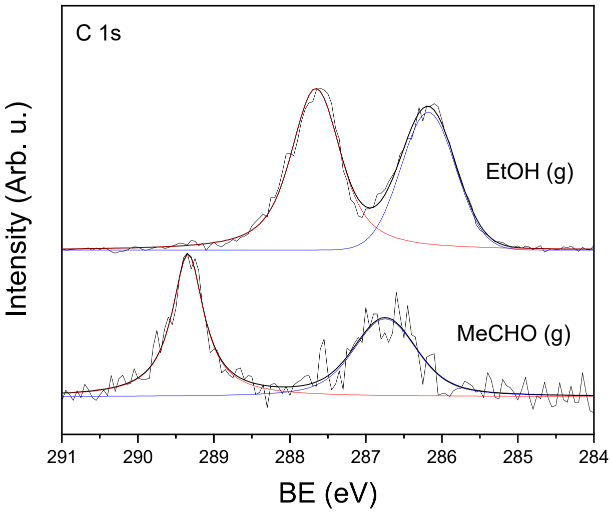

Figure S3. Reference gas-phase C 1s NAP-XPS spectra acquired from the O_2/EtHO and O_2/MeCHO gas mixtures.

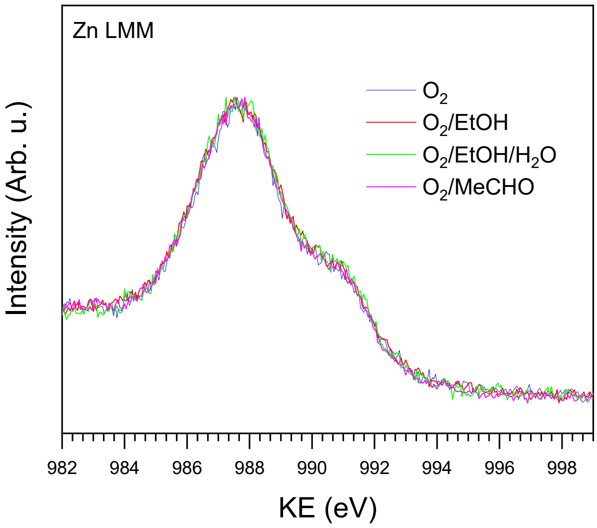

Figure S4. NAP-XPS spectra NAP-XPS spectra of the Zn LMM acquired from the ZnO NRs based sensor in the presence of O_2, O_2/EtOH, O_2/EtOH/H_2O and O_2/MeCHO at 327 °C.
